# A Prospective Multicenter Registry of Patients Undergoing Hysteroscopic Morcellation of Uterine Polyps and Myomas

**DOI:** 10.1089/gyn.2016.0008

**Published:** 2016-12-01

**Authors:** Michael D. Scheiber, Serena H. Chen

**Affiliations:** ^1^ Institute for Reproductive Health, Cincinnati, OH.; ^2^ Institute for Reproductive Medicine and Science at Saint Barnabas Medical Center, Livingston, NJ.

**Keywords:** intrauterine morcellation, hysteroscopic morcellator, endometrial polyp, submucous myoma

## Abstract

***Background:*** Hysteroscopic morcellation removes uterine pathology under direct visualization with continuous real-time tissue fragment removal. ***Objective:*** The aim of this study was to explore the feasibility of hysteroscopic morcellation across a diverse set of facilities, including both surgical and office-based settings. ***Design:*** This was a prospective, single-arm, multicenter registry development (Canadian Task Force classification II-3). ***Materials and Methods:*** Thirty-four U.S. obstetrics and gynecology facilities enrolled subjects into the registry. Inclusion criteria were women ages 18–65 with indications for hysteroscopic myomectomy and/or polypectomy who were treated with the MyoSure® Hysteroscopic Tissue Removal System (Hologic Inc., Marlborough, MA). Intrauterine lesion type/size and removal parameters, adverse events (AEs), and physician satisfaction ratings were recorded. ***Results:*** A total of 559 pathologies (187 fibroids; 372 polyps) were removed from 278 registered subjects (mean age: 43.9 ± 9.0 years), with 250 procedures (89.9%) performed in an ambulatory surgery center or hospital outpatient setting and 28 (10.1%) in a gynecologic office setting. Most patients (*n* = 206, 74.1%) were treated for abnormal uterine bleeding, and 42 (15.1%) were treated for infertility. Mean fibroid diameter was 2.2 ± 1.2 cm. Mean polyp diameter was 1.3 ± 1.0 cm. Overall mean percentage of pathology removed was 95.4% (polyps 99.3%, fibroids 86.8%). Five AEs included four incidents of blunt cervical trauma and a single postoperative case of pedal edema; all were considered mild and resolved spontaneously. Postprocedure surveys indicated that 95% of reporting physicians were “satisfied” or “highly satisfied” with device performance. ***Conclusions:*** Hysteroscopic morcellation of intrauterine pathology was accomplished safely with a high degree of physician satisfaction in 278 patients treated in diverse healthcare settings that are reflective of general community practice in the United States. (J GYNECOL SURG 32:318)

## Introduction

Approximately 30%–50% of abnormal uterine bleeding (AUB) is caused by intracavitary polyps and submucous myomas.^1,2^ There is compelling evidence that removing these growths provides excellent resolution of symptoms.^1–3^ In addition to dilation and curettage and polyp forceps, operative hysteroscopy is among the most common minimally invasive surgical treatments for intrauterine polyp and myoma removal. In electrothermal hysteroscopy, a resectoscope equipped with a radiofrequency-activated cutting loop or an electrical bipolar loop is used to remove intrauterine pathology.^4–6^ Tissue fragments resected from either the polyp or fibroid remain in the distended uterus until manually removed with graspers. As the surgery proceeds, these free-floating tissue pieces can obscure the surgeon's view of the operating site, thereby hindering the procedure. In addition, electrothermal resectoscopy carries a risk of causing inadvertent thermal damage to healthy endometrium that surrounds lesions.^7^ Newer technologies are designed to avert these limitations.

Intrauterine morcellation is a newer approach to operative hysteroscopy that removes uterine pathology under direct visualization with continuous real-time tissue fragment removal. Morcellation instruments use a rotating blade to cut tissue and simultaneously remove resected fragments with uninterrupted irrigation and vacuum aspiration. This approach does not introduce an electrical current inside the uterus and has no risk of potential thermal damage to healthy endometrium.

Compared to resectoscopy, hysteroscopic morcellation reduces operative time for removing uterine lesions. Hamerlynck et al.^8^ reported mean operating times for hysteroscopic morcellation of 34 myomas and 278 polyps at 18.2 and 6.6 minutes, respectively, without complications. Rubino and Lukes^9^ reported a mean resection time of <3 minutes (42 myomas and 76 polyps combined) in 74 patients who underwent morcellation for AUB. Mean pathology removal was nearly complete for both polyps (99.9%) and myomas (95.9%), and patients reported significantly reduced symptoms and improved quality of life (QoL) at postoperative 12 months. In a randomized controlled trial (RCT) of residents-in-training, morcellation reduced total operating time for polyp and myoma removal by 72% and 61%, respectively, versus conventional resectoscopy.^10^ Thus, hysteroscopic morcellation removes intrauterine lesions faster than conventional resectoscopy techniques.

Hysteroscopic myomectomy with an electrothermal loop is conventionally performed in the operating room with the patient under general anesthesia. Researchers have begun assessing the performance of hysteroscopic morcellation in an office versus an ambulatory setting. For instance, Lukes et al^11^ reported results of 5 institutions that successfully removed a total of 42 polyps and 16 myomas in patients treated in an ambulatory surgical center (ASC, *n* = 20 patients) or an office-based setting (*n* = 20 patients). Rubino and Lukes^9^ reported similar outcomes for 32 patients randomized for treatment in an ASC versus 42 patients treated in an office-based setting, with no significant differences in pain scores, adverse events (AEs), patient satisfaction, percentage of pathology removed, or 12-month QoL improvement between ASC and office sites.

Although studies have established the feasibility of hysteroscopic morcellation for polyp and myoma removal in general, most have been restricted to reporting experiences from either a single site or a small number of research-focused institutions. To investigate further the safety and efficacy of hysteroscopic morcellation of intrauterine polyps and myomas in varied community healthcare settings, prospective multicenter studies with larger patient populations are required. To address this need, the current authors established a prospective multicenter registry to assess outcomes better of hysteroscopic morcellation procedures performed in both ASC/hospital outpatient department (HOPD) and office-based settings.

## Materials and Methods

Study subjects had been prospectively registered and enrolled in a multicenter registry. The registry enrolled women ages 18–65 with indications for hysteroscopic myomectomy and/or polypectomy. Inclusion criteria for this study were identification of intrauterine pathology via ultrasound, saline-infusion sonography, or hysteroscopic examination, with polyps of any size and/or submucosal myomas ≤6 cm in diameter. Exclusion criteria were pregnancy, IUD *in situ* at the time of the procedure, current use of anticoagulant or antiplatelet medication in addition to low-dose aspirin, active pelvic inflammatory disease or pelvic/vaginal infection, known or suspected coagulopathy or bleeding disorder, increased fluid-overload risk (e.g., history of predisposing cardiac, hepatic, or renal dysfunction), or other comorbid condition(s) that, in the opinion of the investigator, could limit the ability to participate or affect the scientific integrity of the study.

Written informed patient consent was obtained from each subject prior to study enrollment. To minimize selection bias, all subjects were enrolled who met the inclusion criteria and consented to take part in the study. All procedures were performed with institutional review board approval at each facility. The study protocol adhered to the tenets of the Declaration of Helsinki and was Health Insurance Portability and Accountability–compliant. Procedures were performed in either an ASC/HOPD, or an office setting.

**FIG. 1.**
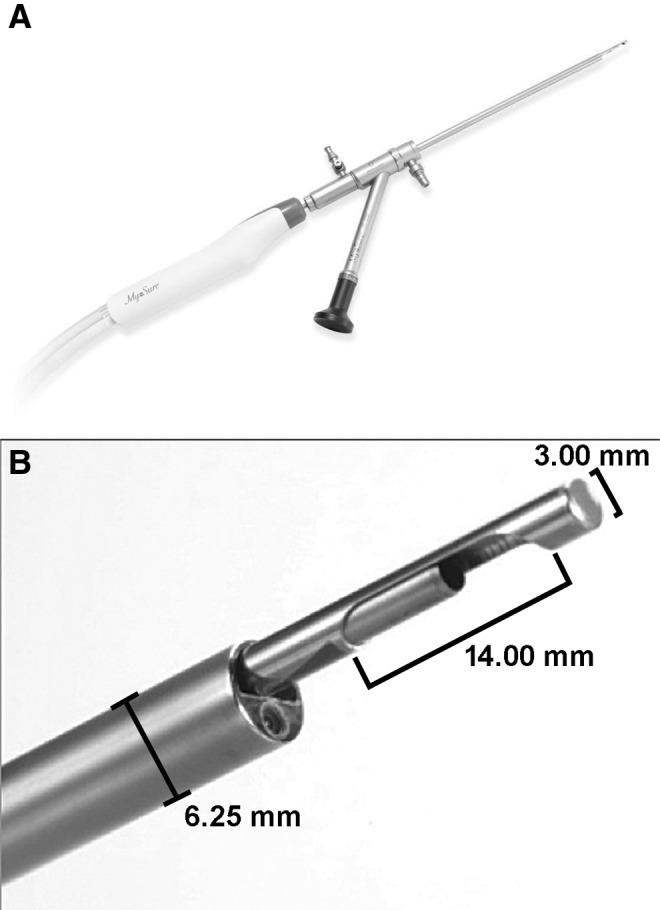
The MyoSure® Hysteroscopic Tissue Removal System (Hologic Inc., Marlborough, MA) includes **(A)** the 3.00-mm-diameter cutting device that is inserted through the system's small-bore, 6.25-mm-outer diameter (19-French equivalent) hysteroscope housing or other conventional surgical hysteroscope sheaths (typically 7–10 mm, or 21–30-French equivalent, in outer diameter). (**B)** Enlarged view of the side-facing cutting window. Under hysteroscopic guidance, the standard unit's cutting blade contacts target tissue through a 14-mm-long and 1.8-mm-deep side channel on the morcellator distal shaft. Foot pedal-controlled activation retracts the blade guard covering the window and engages the stainless steel blade's dual cutting motion, rotating at up to 8075 rpm while oscillating at 3 cycles/second. The system is available in standard, smaller (LITE), and larger (XL) sizes, with slightly different cutting window dimensions and tissue-removal rates. The standard mid-sized device that was used in >90% of procedures is shown.

All morcellation procedures were performed using the MyoSure® Hysteroscopic Tissue Removal System (Hologic Inc., Marlborough, MA).^12^ This device has a side-facing cutter window housing a 2.5-mm-diameter cutting blade, presenting with an outer bevel that rotates at 8075 rpm and oscillates at 3 cycles/second, within a 3-mm outer tube. The system includes a tissue trap that is connected to the tissue removal device through a regulated vacuum (Fig. 1). Constant regulated isotonic electrolyte solution irrigation maintains target intrauterine distension pressure, with continuous aspiration of resected tissues that are suitable for histopathologic analysis.

Age, height, weight, body mass index (BMI), and menopausal status were recorded for each patient. In addition, the researchers documented the number, size, and location of all pathologies (fibroids and polyps), and the primary reason for treatment for each patient. The primary efficacy endpoint was the percentage of lesions removed. Ancillary endpoints included total procedure time, total cutting time, fluid deficit, and the necessity for mechanical cervical dilation. A 5-point Likert scale evaluated physician postoperative satisfaction with the morcellation system, with a score of 5 indicating “very satisfied,” and a score of 1 indicating “very dissatisfied.”

The safety endpoint assessed the incidence of AEs observed prior to patient discharge from the recovery room of the treatment facility or reported by the patient after discharge. Study physicians were requested to report the occurrence of specific potential AEs, which were cramping, nausea, pain, hemorrhage, pulmonary distress, cervical tearing, fibroid recurrence, vaginal bleeding, vomiting, fluid overload, infection, reaction to anesthetic agents, and uterine perforation

Patient characteristics and results of procedures performed in an office setting were compared with those performed in ASCs. Continuous variables were compared using *t*-tests. Categorical data and variables recorded as proportions were compared using Fisher's exact test with 2 × 2 contingency tables. Analyses software included Excel (Microsoft Corp., Bellingham, WA) and Prism, version 5.0 (GraphPad Inc., San Diego, CA). *p*-Values <0.05 were considered indicative of statistically significant differences between comparator groups. Data were analyzed using a modified intention-to-treat basis, and were used to assess all patients who satisfied study enrollment criteria.

## Results

A total of 41 investigators at 34 different institutions treated a total of 278 patients. The participating institutions were diverse with respect to both facility type (7 obstetrics/gynecology offices, 15 nonacademic hospitals/ASCs, 12 academic hospitals/ASCs) and U.S. geographic location (5 Northeast, 7 Midwest, 13 South, and 9 West). Of 278 total subjects, 250 were treated in an ASC or HOPD setting, and 28 were treated in a gynecology office setting (Table 1). The average number of patients treated by each physician was 8.1 (range: 1–20). The proportion of patients who self-identified as non-Caucasian minority was higher in the ASC/HOPD setting (46%) than for patients treated in physician offices (21%; *p* = 0.0156). The percentage of patients treated for a known polyp, fibroid, or other intrauterine mass was higher in the office setting (39%) versus the ASC/HOPD (14%; *p* = 0.0020). Most patients (73.4%) were premenopausal. Two hundred and six patients (74.1%) were treated for AUB, and 42 (15.1%) were treated for infertility. Additional information regarding patient demographics and physician-reported indications for treatment is provided in Table 1.

**Table 1.** Patient Information by Treatment Site Type

*Baseline demographics, mean ± SD*	*All sites(*N* = 278)*	*Office(*n* = 28)*	*ASC/HOPD(*n* = 250)*	p-Value^*^
Age, years	43.9 ± 9.0	45.8 ± 10.4	43.7 ± 8.8	0.2412
Height, cm	163.8 ± 11.7	164.3 ± 6.1	163.6 ± 12.2	0.7449
Weight, kg	79.4 ± 22.1	72.9 ± 17.6	80.1 ± 22.4	0.1012
BMI, kg/m^2^	29.5 ± 8.2	27.0 ± 6.4	29.8 ± 8.4	0.1676
Race, n (%)				
American Indian/Alaska First-Nation	2 (0.7)	0 (0)	2 (0.8)	0.9999
Asian	11 (4.0)	0 (0)	11 (4.4)	0.6098
Black/African American	63 (22.7)	2 (7)	61 (24.4)	0.0537
Hispanic/Latina	42 (15.1)	4 (14)	38 (15.2)	0.9999
Native Hawaiian/Pacific Islander	2 (0.7)	0 (0)	2 (0.8)	0.9999
White	158 (56.8)	22 (79)	136 (54.4)	**0.0156**

*Menopausal status,* n *(%)*	*All sites*	*Office*	*ASC/HOPD*	
Premenopausal	204 (73.4)	18 (29)	186 (74.4)	0.2636
Perimenopausal	25 (9.0)	2 (7)	23 (9.2)	0.9999
Postmenopausal	49 (17.6)	8 (29)	41 (16.4)	0.1092

*Reasons for treatment*^†^, n *(%)*	*All sites*	*Office*	*ASC/HOPD*	
AUB	206 (74.1)	19 (68)	187 (74.8)	0.4949
Infertility	42 (15.1)	2 (7)	40 (16.0)	0.2755
Known polyp/fibroid/uterine mass	46 (16.5)	11 (39)	35 (14.0)	**0.0020**
Postmenopausal bleeding	16 (5.7)	2 (7)	14 (5.6)	0.6684
Other^‡^	9 (3.2)	4 (14)	5 (2.0)	**0.0074**

^*^ Office versus ASC/HOPD.

^†^ Physicians might have reported more than one reason for treatment per patient.

^‡^ Other: dysmenorrhea; anemia; enlarged endometrial stripe; breast-cancer history; recurrent pregnancy loss; and cavity-filling defect.

SD, standard deviation; ASC/HOPD, ambulatory surgery center/hospital outpatient department; BMI, body mass index; AUB, abnormal uterine bleeding.

*Bold* indicates *p*-values < 0.05.

The 559 pathologies removed from the current cohort included 187 fibroids (33.5%) and 372 polyps (66.5%; Table 2). Mean fibroid diameter was 2.2 ± 1.2 cm (range: 0.3–5.5 cm). Mean polyp diameter was 1.3 ± 1.0 cm (range: 0.1–7.0 cm). When comparing pathologies from patients treated in the office versus ASC/HOPD setting, there were no significant differences in lesion type or size. Fibroids were most commonly located in the anterior section of the uterus (26.2% of the total). Polyps were most commonly located in the posterior uterus (24.2% of the total).

**Table 2.** Intrauterine Lesion Parameters, by Treatment Site Type

*Parameter*	*All sites(*N* = 278 patients)*	*Office(*n* = 28)*	*ASC/HOPD(*n* = 250)*
Lesion traits^*^, mean ± SD			
Lesions/subject, n	2.0 ± 1.1	2.0 ± 1.2	2.0 ± 1.1
Fibroids/subject, n	0.7 ± 1.0	0.8 ± 1.1	0.7 ± 1.0
Polyps/subject, n	1.3 ± 1.2	1.2 ± 1.3	1.4 ± 1.2
Fibroid diameter, cm	2.2 ± 1.2	1.9 ± 1.2	2.2 ± 1.2
Polyp diameter, cm	1.3 ± 1.0	1.4 ± 0.8	1.3 ± 1.0

*Fibroid location, n (%)*	*All sites*	*Office*	*ASC/HOPD*
Anterior	49 (26.2)	6 (27)	43 (26.1)
Posterior	40 (21.4)	4 (18)	36 (21.8)
Fundal	36 (19.3)	3 (14)	33 (20.0)
Lower uterine segment	15 (8.0)	2 (9)	13 (7.9)
Lateral left	18 (9.6)	2 (9)	16 (9.7)
Lateral right	29 (15.5)	5 (23)	24 (14.5)
Total	187 (100%)	22 (100%)	165 (100%)

*Polyp location, n (%)*	*All sites*	*Office*	*ASC/HOPD*
Anterior	63 (16.9)	1 (3)	62 (18.3)
Posterior	90 (24.2)	15 (45)	75 (22.1)
Fundal	65 (17.5)	2 (6)	63 (18.6)
Lower uterine segment	42 (11.3)	3 (9)	39 (11.5)
Lateral left	48 (12.9)	4 (12)	44 (13.0)
Lateral right	64 (17.2)	8 (24)	56 (16.5)
Total	372 (100%)	33 (100%)	339 (100%)

^*^ No lesion trait was statistically different between office versus ASC/HOPD settings (*p* > 0.05). Statistics were not performed to compare frequency of lesion intrauterine locations between groups.

ASC/HOPD, ambulatory surgical center/hospital outpatient department; SD, standard deviation.

The overall mean percentage of pathology removed was 95.4% (Table 3). By lesion type, 99.3% of polyps and 86.8% of fibroids were removed. For all subjects, the median reported cutting time was 2.0 minutes (range: 1.0–63.3 minutes), and median fluid deficit was 120 cc (range: 0–2800 cc; Table 3). When surveyed postprocedure, 95% of responding physicians reported they were either “satisfied” or “highly satisfied” with device performance.

**Table 3.** Operative Parameters and Observations

Parameter^*^	*All sites*	n	*Office*	n	*ASC/HOPD*	n	*p-Value*^†^
% Pathology removed, by patient	95.4 ± 13.2%	278	96.8 ± 14.1%	28	95.2 ± 13.1%	250	0.5436
% Polyp removed, by lesion	99.3 ± 5.8%	331	99.3 ± 6.1%	27	99.9 ± 0.4%	304	0.0900
% Fibroid removed, by lesion	86.8 ± 24.1%	157	94.8 ± 17.6%	18	85.8 ± 24.7%	139	0.1368
Adverse events, % of patients	1.8%	278	3.6%	28	1.6%	250	0.4143
Fluid deficit, cc	287 ± 453	234	238 ± 666	17	291 ± 446	217	0.6510
Resection time, min	6.0 ± 9.0	229	8.9 ± 15.6	21	5.8 ± 8.1	208	0.0978
Time in PACU, min	55.4 ± 37.1	207	36.8 ± 24.7	16	57.0 ± 37.5	191	**0.0263**
Physician satisfaction score 4–5, %	95%	278	89%	28	96%	250	0.1470
Anesthesia type, *n* (%)							
Oral sedation	6 (2.2%)		5 (18%)		1 (< 1%)		**< 0.0001**
Cervical block	41 (14.7%)		15 (54%)		26 (10.4%)		**< 0.0001**
IV sedation	68 (24.5%)		17 (61%)		51 (20.4%)		**< 0.0001**
General anesthesia	196 (70.5%)		2 (7%)		194 (77.6%)		**< 0.0001**

^*^ Values provided as mean ± standard deviation, or *n* (%), as appropriate.

^†^ *p*-Values compare office to ASC/HOPD.

ASC/HOPD, ambulatory surgical center/hospital outpatient department; min, minutes; PACU, post-anesthesia care unit; IV, intravenous.

*Bold* indicates *p*-values < 0.05.

Of 245 subjects for whom cervical dilation information was recorded, mechanical dilation was performed for 215 patients (87.7%), and mean dilation was 0.9 ± 0.5 cm (range: 0.3–2.8 cm; not shown). General anesthesia was administered in a greater proportion of patients treated in the ASC/HOPD setting (78%) versus the office setting (7%; Table 3). Office-based procedures used oral and intravenous sedation and cervical blocks more frequently than ASCs and HOPDs (*p* < 0.0001 for each anesthesia type). Mean postoperative recovery time was 54% longer in the ASC/HOPD setting (57 minutes) than for procedures performed in the office (37 minutes; *p* = 0.0263).

Five AEs were reported in the 278 subjects; 4 patients had mild cervical trauma and 1 patient experienced a moderate case of postoperative pedal edema. All AEs were considered mild and resolved spontaneously.

## Discussion

The primary endpoint of this study was pathology removal. This study showed a high percentage of removed pathology (95.4%) in all patients using hysteroscopic morcellation, with no significant difference between office and ASC/HOPD settings. While postprocedure clinical symptoms were not recorded in this registry, a previous study demonstrated favorable improvement in 1-year clinical symptom and QoL for patients whose fibroids were removed incompletely.^9^ Importantly, residual fibroid tissue left behind after incomplete myoma resection spontaneously regresses in >50% of AUB patients with incomplete primary resection, primarily during the first 3 months after intervention, and often in entirety.^13^ Thus, one can expect observable normalization of endometrial morphology and function after uterine lesion morcellation, including in women with limited amounts of residual pathology.

Reported AE rates in this study were low and all AEs were considered mild. This is consistent with previous findings by others, including Emanuel,^14^ who noted that morcellation does not use electrocoagulation and that there is no lateral thermal or electrical energy spread. Instead of thermal coagulation, hemostasis after morcellation occurs by spontaneous myometrial contraction. In addition, because hysteroscopic morcellation removes lesion fragments automatically while inserted into the uterus, the procedure is associated with a decreased number of device transcervical introductions, thereby minimizing the likelihood of cervical trauma and uterine perforations. Researchers who conducted an RCT of residents-in-training reported that morcellation reduced total operating time for polyp and myoma removal by 72% and 61%, respectively, versus conventional resectoscopy.^10^ Part of this time saving was attributed to the reduced number of device insertions during procedures, ranging from 1–2 insertions with morcellation versus 3–50 with conventional resectoscopy. These factors combined to result in fewer complications with hysteroscopic morcellation, compared to electrosurgical resection. Likewise Smith et al.^15^ found that morcellation was significantly quicker than electrosurgical resection for hysteroscopic polypectomy, and was less painful, more acceptable to women, and more likely to remove endometrial polyps completely.

Nearly 50% of hysteroscopic complications are related to difficulty in traversing the cervix.^16^ The MyoSure operative resectoscope has an outer diameter of 6.25 mm (19-French), versus the 7–10-mm diameter (21–30-French) of most surgical sheaths. Small-bore hysteroscopes require less cervical dilation for uterine access, and are associated with decreased patient pain and discomfort, fewer procedural complications, and greater patient satisfaction, for both diagnostic^17^ and surgical^18^ applications.

Operative hysteroscopy is still most commonly performed in surgical settings. The current study had similar favorable clinical outcomes regardless of site of service, thus demonstrating the feasibility of performing hysteroscopic morcellation of uterine pathologies in an office-based setting. This finding is supported by earlier studies that demonstrated favorable results for in-office hysteroscopic morcellation^9^ or other in-office hysteroscopic procedures.^19^ The utility of office-based hysteroscopic morcellation is also supported by a study that demonstrated that low pain scores are achievable using local anesthetic delivered by paracervical-block protocols.^10^ Finally, morcellation devices are compatible with newer fluid-management systems that are more reliable and precise for regulating fluid delivery and retrieval rates, thereby improving patient safety.^19–21^

Subjects in the current study were most commonly treated for AUB or infertility, demonstrating that hysteroscopic morcellation can be used for diverse clinical indications. This registry included >250 women treated at geographically diverse academic and community-based facilities. With >30 office and surgical sites, and 40 investigators of varying levels of experience contributing results, this study provides a current and representative depiction of hysteroscopic morcellation used in routine practice across the United States. This study supports the feasibility of using hysteroscopic morcellation to treat intrauterine pathology in a wide range of clinical settings.

The strong points of this study are its prospective nature, the relatively large overall cohort size, and the diversity in geographic locales and nature of participating healthcare facilities. The primary study limitation was that the single-arm design did not compare outcomes between hysteroscopic morcellation and other uterine pathology treatments, such as resectoscopy. Another limitation was the relatively small size of the office-based cohort arm. Expansion of the currently established registry will complement new and ongoing RCTs in assessing the utility of hysteroscopic morcellation for ameliorating intrauterine pathologies safely in both office and ASC settings.

## Conclusions

This study demonstrated the successful use of hysteroscopic morcellation of uterine fibroids and polyps in >250 patients treated in settings reflective of general community practice in the United States. Hysteroscopic morcellation is a feasible approach for removing intrauterine lesions in both ASC/HOPD and office-based settings, has an excellent safety profile, and provides a high level of physician satisfaction.
